# Supplementary figures and images for: Activation of cryptic splicing in bovine WDR19 is associated with reduced semen quality and male fertility
Source: PLoS Genet. 2020 May 14;16(5):e1008804. doi: 10.1371/journal.pgen.1008804 (PMC7252675; doi:10.1371/journal.pgen.1008804)

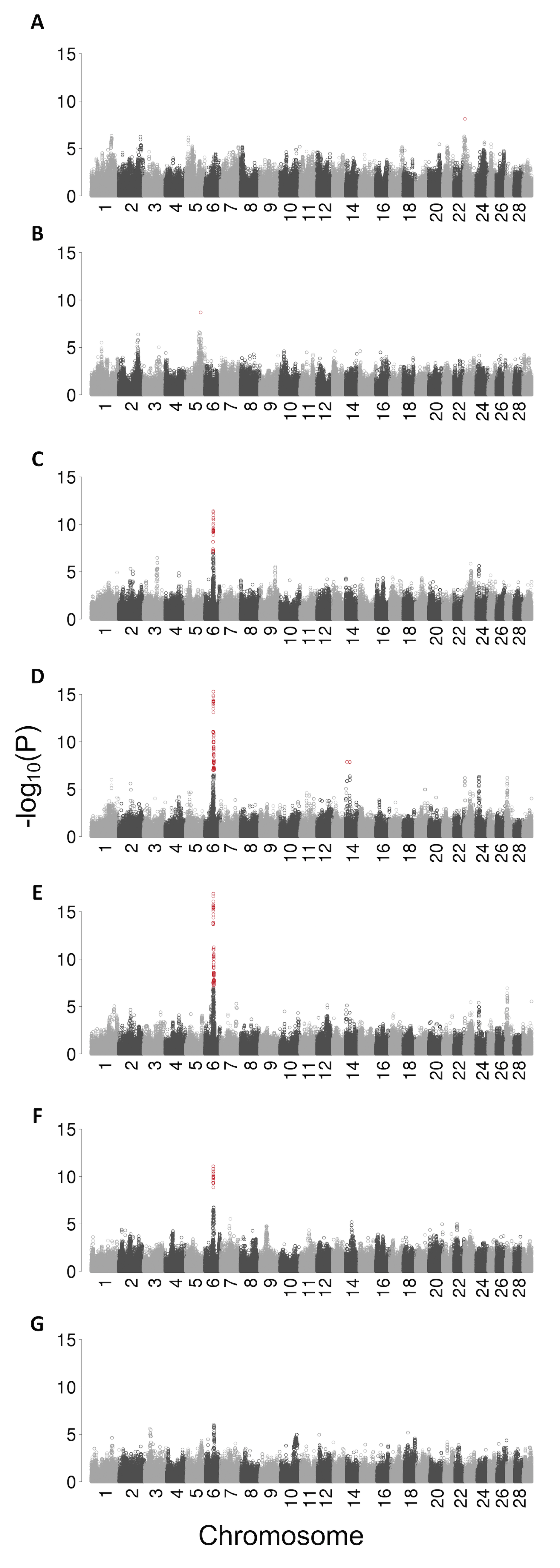

Supplement: S1 Fig — Manhattan plots representing the association (–log10(P)) of haplotypes with (A) ejaculate volume (genomic inflation factor lambda = 1.67), (B) sperm concentration (lambda = 1.29), (C) sperm motility (lambda = 1.35), (D) proportion of sperm with head anomalies (lambda = 1.10), (E) proportion of sperm with tail anomalies (lambda = 1.14), (F) sperm per straw (lambda = 1.22), and (G) bull fertility (lambda = 1.22) assuming an additive mode of inheritance. Red color indicates significantly associated haplotypes (P < Bonferroni corrected significance threshold). (TIF) [file pgen.1008804.s005.tif]

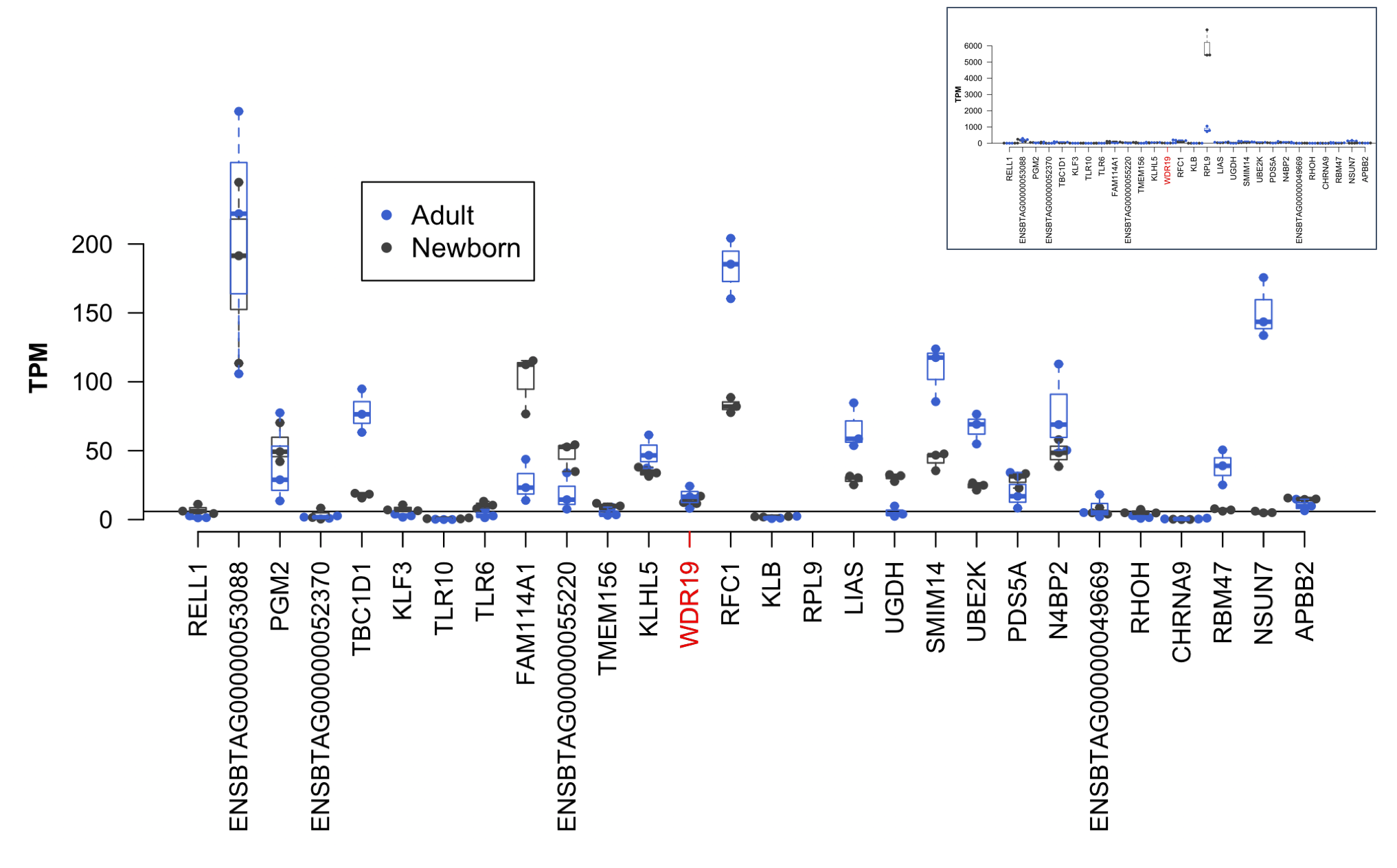

Supplement: S2 Fig — Transcripts per million (TPM) in testis tissue of three mature bulls (grey) and three newborn male calves (black). The horizontal line represents the median expression (5.9 TPM) of 22,372 genes. To improve readability, the expression for RPL is only shown in the inset. (PNG) [file pgen.1008804.s006.png]

A

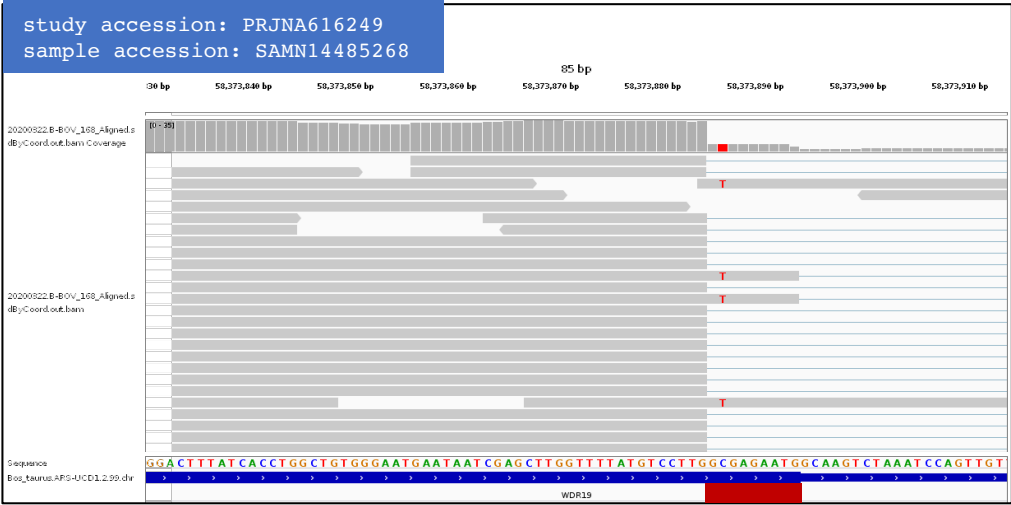

*mt/mt*

B

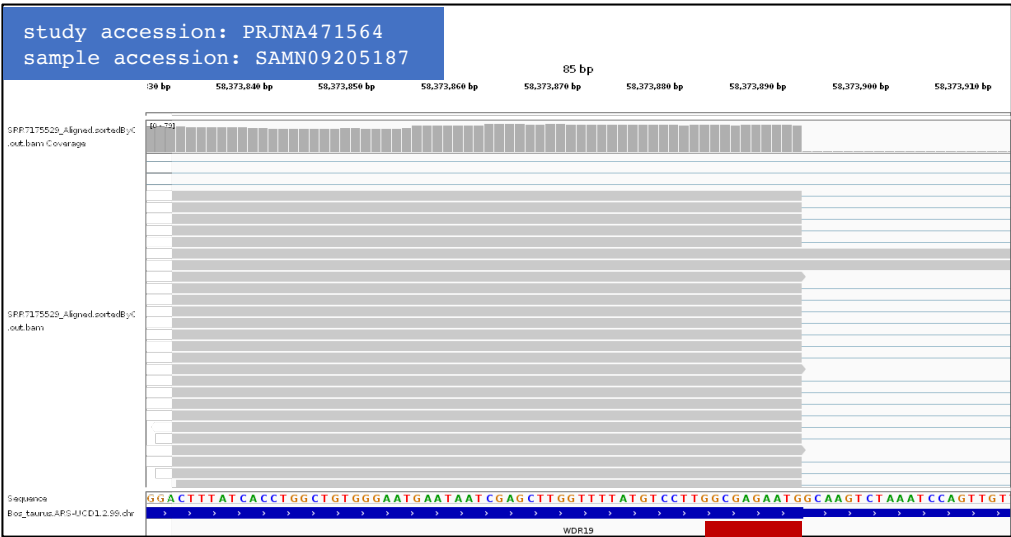

*wt/wt*

C

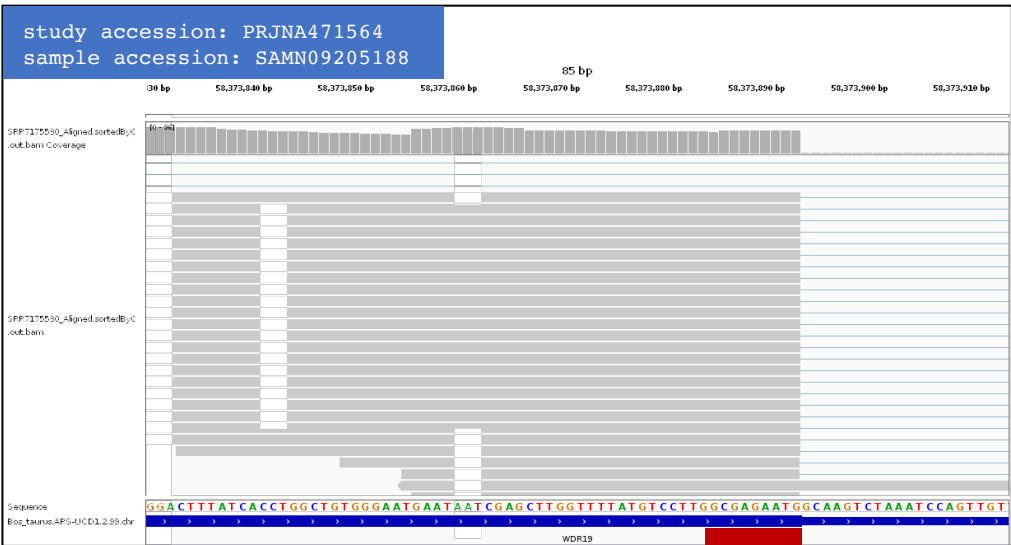

*wt/wt*

Supplement: S3 Fig — Screen captures of IGV outputs from testis RNAseq alignments of a BSW bull (SAMN14485268) homozygous for the mutant (mt) T-allele (A) and two control bulls from the Angus breed (SAMN09205187, SAMN09205188) that are homozygous for the wild-type (wt) C-allele at Chr6:58373887 (B, C). The red bar indicates nine nucleotides that are truncated from exon 12 of WDR19 in the BSW bull (A) due to cryptic splicing activated by the T-allele. A low number of sequence reads corresponding to the wild-type transcript were also detected in the bull homozygous for the BTA6:58373887 T-allele. (PDF) [file pgen.1008804.s007.pdf]

A

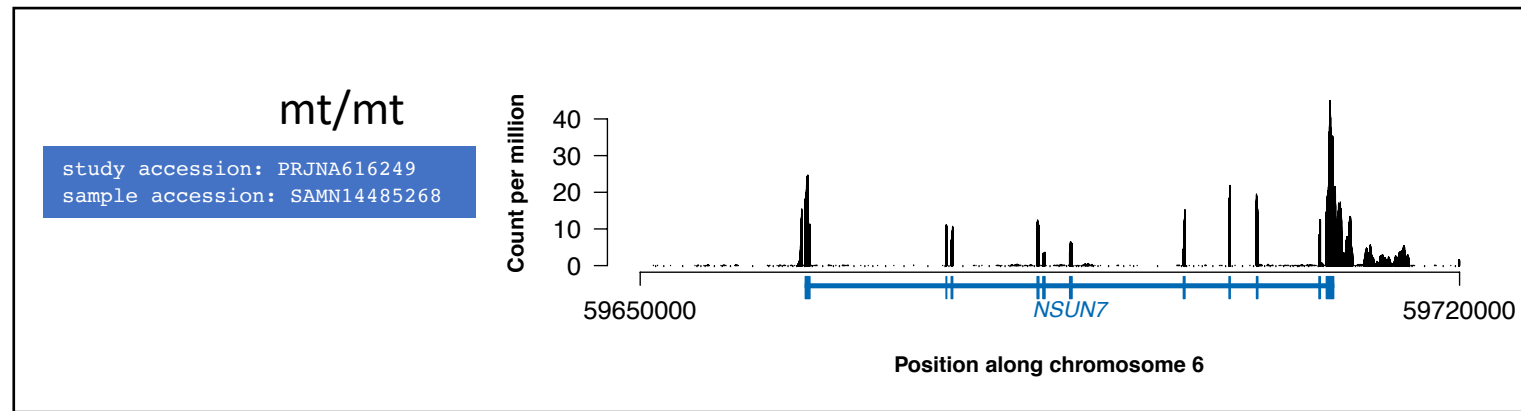

B

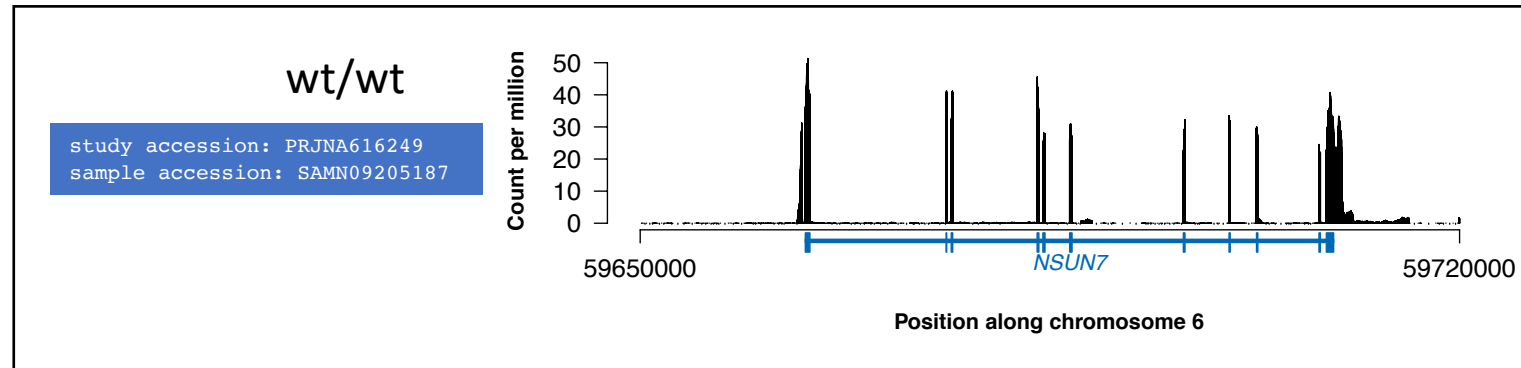

C

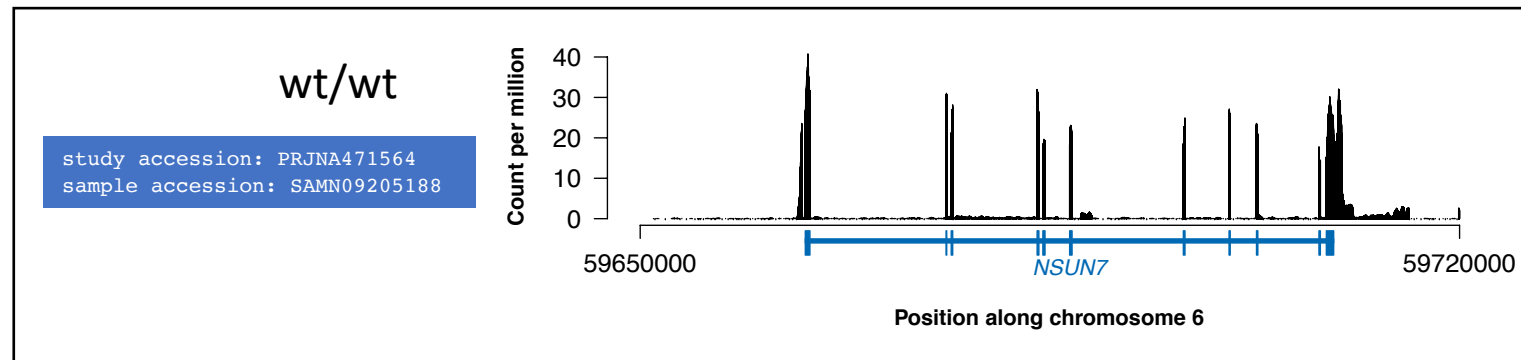

Supplement: S4 Fig — Expression of NSUN7 quantified using testis RNAseq alignments of a BSW bull (SAMN14485268) homozygous for the mutant (mt) T-allele (A) and two control bulls from the Angus breed (SAMN09205187, SAMN09205188) that are homozygous for the wild-type (wt) C-allele at Chr6:58373887 (B, C). The number of reads covering a genomic position was extracted from coordinate sorted BAM files using the mosdepth software and subsequently divided by the total number of reads (in million) mapped to transcripts. (PDF) [file pgen.1008804.s008.pdf]
