# Supplementary material for: Activation of cryptic splicing in bovine WDR19 is associated with reduced semen quality and male fertility
Source: PLoS Genet. 2020 May 14;16(5):e1008804. doi: 10.1371/journal.pgen.1008804 (PMC7252675; doi:10.1371/journal.pgen.1008804)
Supplement: Attachment — Submitted filename: rebuttal_letter.pdf [file pgen.1008804.s011.pdf]

Reviewer #1:

Reviewer #1: Based mostly on GWAS and impressive genomic analyses, the authors identified a synonymous SNP in WDR19 gene of Brown Swiss cattle. They present circumstantial evidence that the frequent minor allele of this SNP activates cryptic splicing resulting with a deleterious effect on semen quality and male fertility. If true, their work presents most-valuable evidence for the importance of this gene in male fertility. Besides their prepublication in Biorxiv, their report is the first one to demonstrate this importance and thus would be of interest to a wide-readership. Yet, as indicated in Major Concerns, another strong candidate gene for male fertility is within the haplotype sharing the 2.38 Mb segment of extended autozygosity. The authors should consider further experimentation to prove that expression of this other gene is intact in homozygotes for the deleterious haplotype.
Author: Following the reviewer's suggestion, we collected RNA sequencing data (63 million reads) of one bull homozygous for the BTA6:58373887 T-allele in order to investigate NSUN7 expression and the abundance of the two WDR19 isoforms. The data have been deposited at the NCBI SRA (study accession: PRJNA616249; sample accession: SAMN14485268). Please find more details below.

Reviewer #1: 1. The authors ignore substantial literature related to the Nsun7 gene, which resides in the critical segment that they identified. This gene that was initially identified in infertile male mice (Harris et al., 2007, Biol Reprod. 77:376-82) has a deletion mutation associated with sperm motility defect in infertile men (Khosronezhad et al., 2015, J Assist Reprod Genet. 32:807-15). Unlike WDR19, NSUN7 gene is highly expressed in the authors' RNA-seq meta-analysis of the testis.
Author: Indeed, we did not focus on this gene because we did not detect any variants nearby or within NSUN7 that were compatible with the inheritance pattern of the BTA6 QTL. We now state in the results section (line 207ff): "Of these genes, 15 and 14 are expressed at medium to high levels (> 10 transcripts per million (TPM)) in the testes of newborn and mature males, respectively (Supporting File 3) including NSUN7 encoding NOP2/Sun RNA Methyltransferase Family Member 7. Loss-of-function alleles in NSUN7 have been associated with low sperm motility and impaired male fertility in mice and humans [33],[34].". We also write (line 235ff) "We did not detect any sequence variants nearby the *NSUN7* gene neither in coding nor in non-coding sequences that were compatible with the inheritance pattern of the top haplotype (Figure 3C, Supporting File 5).". Moreover, as suggested by the reviewer, we used RNA sequencing data of one bull homozygous for the BTA6:58373887 T-allele to investigate the expression of NSUN7. These data are presented in a newly compiled Supplementary figure. Specifically, we write (line 312): "The expression of *NSUN7* was intact in the BSW bull homozygous for the BTA6:58373887 T-allele (Supporting File 8).

Reviewer #1: 2. Brown Swiss cattle are some what under represented in the Short Read Archive (SRA). Yet, six RNA-seq submissions from skin samples (ERX1545689-94) are available. Using 32 bp probes for the three possible sequences of relevant splice junction, I BLASTN searched these SRA submissions concluding that it is spliced as expected (179 hits found) with no indication of the frequent mutated allele. Thus, performing such RNA-seq experiment using a testis tissue sample from a homozygote for the haplotype associated with infertility would allow to simultaneously asses if NSUN7 expression is intact, while WDR19 is not. This evidence is much needed to support the authors' claim.
Author: We downloaded the six RNA-seq submissions mentioned by reviewer#1 and aligned them to the reference sequence using the STAR aligner. They all are indeed

homozygous for the BTA6:58373887 C-allele (wild type). As suggested by the referee, we collected RNA sequencing data (63 million reads) of one bull homozygous for the BTA6:58373887 T-allele in order to investigate NSUN7 expression and the abundance of the two WDR19 isoforms. The data have been deposited at the NCBI SRA (study accession: PRJNA616249; sample accession: SAMN14485268). These data confirmed our RT-PCR findings (also presented in a new Supporting File 7) and enabled us to estimate the abundance of the two WDR19 isoforms. We write in the main part (line 306 ff): "In order to quantify the abundance of the two WDR19 isoforms, we generated 63 million 150 bp sequencing reads from RNA prepared from testicular tissue sampled from one BSW bull (sample accession number SAMN14485268) homozygous for the BTA6:58373887 T-allele. Of 29 RNA-seq reads that overlap the junction of exons 12 and 13, 5 and 24 correspond to the wild-type and mutant transcript, respectively, indicating that the wild-type and mutant isoforms are expressed at a ratio of 1:5 (Supporting File 7).". We also added a paragraph describing the processing of RNA-seq data to the Material and Methods section (line 738 ff).

Reviewer #1: Minor Comments:
Line 79: "pathopysiology" better "pathophysiology".
Lines 101 & 202: " mimimum"/"miminum" should be "minimum".
Line 286: "WD repeat-contaning" should be "WD repeat-containing"
Line 472: "protein wich" should be "protein, which".
Line 662: "Strcutural" should be "Structural".
Author: Thank you very much for catching the typos. We fixed all of them

Reviewer #2
Reviewer #2: Hiltpold et al. have identified a cryptic exonic splice variant in the WDR19 gene in cattle and in homozygous condition resulted in lower semen quality and lower male fertility. They used a haplotype-based association mapping followed by searching the candidate variants using genome sequence data. The variant was validated in independent populations. Western blot analysis was done to study the protein expression, which further confirmed the functionality of this variant. The manuscript is well written and easy to follow. I have only a few minor comments.
Author: Thank you for your valuable comments. We addressed all of them in the revised version.

Reviewer #2: Introduction part is too long and may be shortened.
Author: Agreed. We shortened the introduction by ~30%, and its revised version is now more concise.

Reviewer #2: The authors described the WDR19 variant had recessive gene effect. However, markedly reduced WDR19 protein expression in testicular tissue of heterozygous and homozygous bulls were observed (L475). Therefore, it follows an underlying liability model rather than a recessive model, where heterozygous individuals are expected to be similar to the wild type homozygotes.
Author: We considered your suggestion in the discussion. Specifically, we write (line 484ff): "Factors controlling splice site selection may contribute to the marked phenotypic variability in semen quality in bulls homozygous for the BTA6:58373887 T-allele. The presence of a low amount of wild-type WDR19 protein seems to be sufficient to produce normal sperm. However, semen quality of individuals that exceed a certain threshold of mutant protein or

undercut a certain threshold of wild-type protein might have a higher proportion of abnormal sperm, thus resulting in lower fertility.”. Moreover, we clarify that protein abundance was less in homozygotes than heterozygotes (l.327))

Reviewer #2: The heritability for tail and head anomalies were zero or close to zero, though both had moderate repeatability estimates. This may be discussed.
Author: We now highlight the difference between repeatability and heritability. We write in the revised results part (l. 139): “The repeatability of the semen traits was between 0.38 and 0.61, but the heritability was clearly lower indicating that permanent environmental effects markedly affect semen quality” - and in the revised discussion part (l. 376ff): “The repeatability was higher than heritability for all traits analysed indicating that the semen quality of AI bulls is markedly influenced by permanent environmental effects that may also capture non-additive genetic effects [46], which agrees with previous findings in boars and bulls [9],[47].”

Reviewer #2
L435: date -> data
L470: wich -> which
L555: Ø?? With such large variation in ejaculate number, the median may be given rather than mean.
L580: Replace ‘either semen quality parameter’ -> a semen quality phenotype.
L590: Please edit the sentence ‘Because the means … analyses’.
L635: (qchisq(1-p,1)’)’
L700: mt and wt are not defined yet.
L1115: (m) -> (mt)
Author: We fixed all of these points raised.
