# Supplementary material for: Activation of cryptic splicing in bovine WDR19 is associated with reduced semen quality and male fertility
Source: PLoS Genet. 2020 May 14;16(5):e1008804. doi: 10.1371/journal.pgen.1008804 (PMC7252675; doi:10.1371/journal.pgen.1008804)
Supplement: Attachment — Submitted filename: rebuttal_letter_R1.pdf [file pgen.1008804.s012.pdf]

Reviewer #1: In the revised version, the authors added an analysis of the important NSUN7 candidate gene concluding that this gene's expression was intact in a homozygote for the male infertility BTA6 QTL. Although, the 5' end of NSUN7 was under-expressed by this homozygote (Supporting File 8, SAMN14485268) comparing to the Angus controls (please correct to SAMN09205187, in panel B, Supporting File 8), I tend to accept their interpretation. Yet, I suggest the use of a more careful phrasing while describing this "intact" expression, introducing explanation why it was lower for the 5' end comparing to the controls. Generally, the authors addressed my concerns and therefore I think that their work would be a valuable contribution to Plos Genetics.
Author: Thank you for your comments. We now write (lines 313-315) "The mRNA expression pattern of *NSUN7* was similar in the BSW bull homozygous for the BTA6:58373887 T-allele and two Angus bulls homozygous for the wild-type C-allele (Supporting File 8).". Moreover, we corrected the mistake in the supporting file and uploaded an adjusted Supporting File 8.

Reviewer #2: The authors have revised the manuscript satisfactorily. I have no further comments.
Author: Thank you.
